# Retention forces between primary and secondary CAD/CAM manufactured telescopic crowns: an in vitro comparison of common material combinations

**DOI:** 10.1007/s00784-021-03928-2

**Published:** 2021-04-08

**Authors:** Martin Schimmel, Moritz Walther, Nadin Al-Haj Husain, Kensuke Igarashi, Julia Wittneben, Samir Abou-Ayash

**Affiliations:** 1 Department of Reconstructive Dentistry and Gerodontology, School of Dental Medicine, University of Bern, Bern, Switzerland; 2 Division of Gerodontology and Removable Prosthodontics, University of Geneva, Geneva, Switzerland; 3 Department of Dental Materials Science, School of Life Dentistry at Niigata, The Nippon Dental University, Niigata, Japan; 4 Section for Digital Implant- and Reconstructive Dentistry [DIRecD], Department of Reconstructive Dentistry and Gerodontology, School of Dental Medicine, University of Bern, Freiburgstrasse 7, 3010 Bern, Switzerland

**Keywords:** Alloy, PEEK, Retention force, Telescopic crown, Wear, Zirconia

## Abstract

**Objectives:**

To analyze the retention forces between primary and secondary telescopic crowns milled from various materials and to compare them with the retention forces between cast telescopic crowns made of precious metal alloys.

**Materials and methods:**

Primary and secondary crowns (*N* = 60; *n* = 10 per group) were fabricated using various material combinations (1: zirconia [ZIR]/polyether ether ketone [PEEK]; 2: titanium grade IV [TI]/PEEK; 3: PEEK/PEEK; 4: non-precious alloy [NPA]/PEEK; 5:NPA/NPA), while precious alloy (PA) was used for the control group (6: PA/PA). The retention forces at 10, 1000, 5000, and 10,000 connection and disconnection cycles and the relative weights were analyzed, applying nonparametric repeated measures ANOVA and post hoc Mann–Whitney and Wilcoxon signed-rank tests (*α* < 0.05).

**Results:**

Globally, significant differences in the retention forces among the materials (*p* < 0.0001), time points (*p* < 0.0001), and wear resistance for the various materials (*p* < 0.0001) were observed. No significant changes in retention forces compared to baseline were observed in groups 2, 4, 5, and 6. A significantly higher weight loss for both primary and secondary crowns was observed in groups 4 and 6.

**Conclusions:**

The material combination in telescopic attachments influences retention forces and wear. Interactions between materials and time were evident, indicating that the change in retention forces differs among the materials. The combinations of milled TI/PEEK and NPA/NPA qualify for further preclinical testing in a more clinically realistic setup, determining a material-specific double-crown design.

**Clinical relevance:**

The design of precious alloy telescopic crowns cannot be directly transferred to other milled material combinations due to different retention behaviors.

## Introduction

In partially edentulous patients, removable partial dentures (RPDs) represent one of the available treatment options for replacing missing teeth, especially when multiple teeth or adherent structures such as hard and soft tissues must be replaced [1]. There are several RPD types, with clasp-retained RPDs being the most commonly applied type worldwide [2]. RPDs can successfully be combined with strategically placed implants, which might be of particular benefit in clinical situations with extended edentulous ridges. However, these tooth-implant-retained RPDs usually compose multiple types of attachments on the abutment teeth and implants. Whether this combination of different attachments affects abutment survival or complication frequencies has not yet been conclusively clarified [3]. Double crowns can be used on both teeth and implants and therefore represent one option to overcome the use of mixed attachments in tooth-implant-supported RPDs. In general, reported abutment and prosthetic survival rates in double-crown-retained RPDs are higher compared to classical clasp-retained RPDs [4].

Double-crown attachments consist of a primary crown, which is directly cemented to an abutment tooth or screwed onto an implant, and a secondary crown, which is incorporated in the denture. Double crowns are frequently applied, especially in Japan and Germany [5–8]. Among the various types of double crowns, telescopic crowns with a parallel- or nearly parallel-walled (0–2°) primary crowns are commonly applied [9].

Typically, the combination of materials in telescopic-crown-retained RPDs composes a metal–metal, zirconia–metal, or metal–polymer contact, which has different surface wear patterns and, therefore, variable resistance to repetitive removal–insertion cycles [9, 10]. Commonly, a hard material that shows high resistance to wear was chosen as the primary crown and a more flexible material as the secondary crown. Historically, primary and secondary crowns were cast from precious or non-precious metal alloys [4]. The time-consuming casting techniques, especially in combination with the use of precious alloys, resulted in high manufacturing costs and consequently in high prices relative to clasp-retained RPDs. Furthermore, the casting of non-precious alloys is a challenging procedure, resulting in varying alloy compositions, surface properties, and consequently different levels of biocompatibility [11]. With the advent of computer-aided design (CAD) and computer-aided manufacturing (CAM) technologies, precision milling of inner and outer crowns has been more and more frequently applied. Consequently, further materials for primary and secondary crowns including zirconia (ZrO_2_), titanium, or high-strength resins such as polyether ether ketone (PEEK) [12–16] have been introduced. Milling primary and secondary crowns from these materials may help reduce human labor and manufacturing costs, and the related financial burden for the patient, of double-crown-retained RPDs [17]. Furthermore, only a single study which has systematically evaluated the evolution of retention forces using various material combinations, especially regarding milled primary and secondary crowns, could be identified [18]. Therefore, the current study aimed to analyze the progression over time of retention forces of various material combinations in milled primary and secondary crowns and to compare it to the golden standard (cast precious alloy primary and secondary crowns) with the same design. The null hypothesis was that the retention forces of various milled primary and secondary crowns will be equal to those of casting precious alloy crowns after 10,000 connection and disconnection cycles, simulating 10 years of use. Furthermore, the loss of mass after the simulated 10-year use was to be measured as a secondary outcome.

## Materials and methods

### Specimen preparation

A total of 1 control and 5 test groups containing 10 specimens each (*n* = 10) were defined, resulting in a total of 60 specimens. The primary crowns of the test groups were made of a non-precious alloy (NPA), zirconia (ZIR), polyether ether ketone (PEEK), or titanium (TI). The secondary crowns were made of NPA or PEEK. Primary and secondary crowns made out of a precious alloy (PA) served as the control group. The specifications of the applied materials and their combinations are given in Tables 1 and 2.

**Table 1:** Overview and composition of applied materials. *PA*, precious alloy; *NPA*, non-precious alloy; *PEEK*, polyether ether ketone; *TI*, titanium; *ZIR*, zirconia

Material	Brand name	Manufacturer	Composition
ZIR	DC Zirkon Premium	Dental Concepts Systems GmbH, Wahlsburg, Germany	
TI	DC Titan Grade 4	Dental Concepts Systems GmbH, Wahlsburg, Germany	Titanium: > 99% Others: < 1%
PEEK	BioHPP	Bredent Group, Senden, Germany	Polyether ether ketone: 80% Inorganic fillers: 20%
NPA	DC NP EXPERT C+B 270	Dental Concepts Systems GmbH, Wahlsburg, Germany	Cobalt: 65% Chrome: 28.5% Molybdenum: 5.5% Others: < 1%
PA	Protor 3	Cendres Metaux, Biel/Bienne, Switzerland	Gold: 68.6% Platinum: 2.45% Palladium: 3.95% Silver: 11.85% Copper: 10.6% Tin: 2.5% Irridium: 0.05%

**Table 2:** Applied material combinations for the fabrication of primary and secondary crowns. *PA*, precious alloy; *NPA*, non-precious alloy; *PEEK*, polyether ether ketone; *TI*, titanium; *ZIR*, zirconia

	Primary inner crown	Secondary outer crown
Test 1 (*n* = 10)	ZIR	PEEK
Test 2 (*n* = 10)	Ti	PEEK
Test 3 (*n* = 10)	PEEK	PEEK
Test 4 (*n* = 10)	NPA	PEEK
Test 5 (*n* = 10)	NPA	NPA
Control (*n* = 10)	PA	PA

**Fig. 1:**
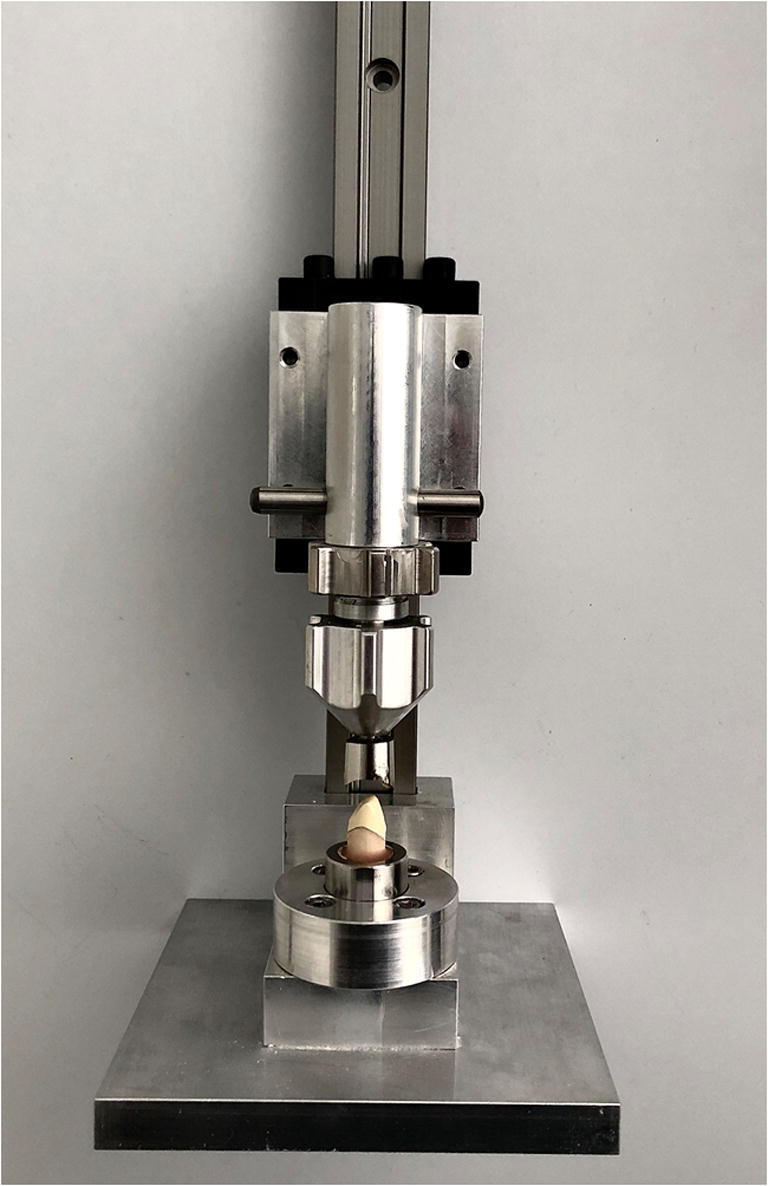
Customized device for standardized base plate fabrication for the artificial tooth and cementing of the primary crown

An artificial typodont (FDI 13, Nissin, Kyoto, Japan) was prepared for a full crown according to the following design: a shoulder of 1.2 mm, axial reduction of 1.2 mm, an axial taper of 6°, and incisal clearance resulting in a final height of 5 mm. Afterward, the prepared tooth was digitized with a laboratory scanner (DW Series 7, Dentalwings, Chemnitz, Germany) and milled (M1; Zirkonzahn, Gais Italy) from polyurethane resin (Try-in III; Zirkonzahn, Gais, Italy) (*n* = 60). The bottom of the tooth had an oval shape with one flattened surface, guaranteeing precise repositioning in a customized device for standardized base plate manufacturing and subsequent luting of the primary crowns to the artificial teeth (Fig. 1). The teeth were embedded in polymethyl methacrylate (PMMA) (Paladur®; Kulzer GmbH, Hanau, Germany) resin base plates.

### Milling procedure

**Fig. 2:**
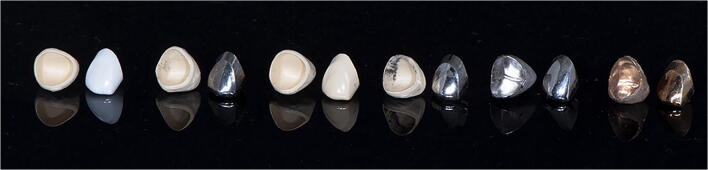
All types of primary and secondary crowns, from left to right: Zirconia/PEEK, non-precious alloy/PEEK, PEEK/PEEK, titanium/PEEK, non-precious alloy/non-precious alloy, and precious alloy/precious alloy

CAD was used for the primary and secondary crowns of the test groups. Subsequently, all crowns were milled using standardized parameters on the same milling machine (DC5; Dental Concept Systems GmbH, Ulm, Germany): a cement gap of 0.02 mm, a horizontal crown border offset of 0.1 mm, and a vertical offset of 0 mm. The wall thickness of all primary and secondary crowns was 0.3 mm to evaluate if all crowns would survive the cyclic connections and disconnection despite their small wall thickness. The retention height was 4.5 mm interdentally and 2 mm buccally/orally, and the taper was 1°. The outer surface of the primary crowns was finished by manual polishing without additional grinding. The polishing was done, following material-specific polishing tools, always by the same master dental technician (Table 3). The inner surface of the secondary crowns was not modified. For the control group, the primary crowns were milled from wax with the same parameters applied to the test groups and subsequently converted to PA crowns using the lost-wax technique. After polishing, the secondary crowns were manually created from wax, copying the digital design of the secondary crowns as well as possible, and subsequently also cast from the same PA. An overview of all types of primary and secondary crowns is presented in Fig. 2. A tertiary structure was designed based on the secondary crown design and milled from a non-precious alloy. The design covered the secondary crowns completely to prevent bending during cyclic loading and included a direct connection to the collet of the test device (Model 5942; Instron France SAS, Elancourt, France).

**Table 3:** Overview of the material-specific polishing steps and instruments. All instruments were from Fa. Bredent, Senden, Germany. *NPA*, non-precious alloy; *TI*, titanium; *PEEK*, polyether ether ketone; *ZIR*, zirconia

	NPA/TI	PEEK	ZIR
Step 1	Ceragum Gummipolierwalze REF PWKGO600	Ceragum Gummipolierwalze REF PWKGO600	Rundbürsten Ziegenhaar REF 35000540
Step 2	Rundbürsten Chungking REF 35000510	Rundbürsten Ziegenhaar REF 35000540	Zi-polish REF 36010025
Step 3	Abraso Starglanz REF 52000163	Lederschwabbel REF 35000660	
Step 4		Abraso Starglanz REF 52000163

### Crown conditioning and cementation

The inner surfaces of the primary crowns and the tertiary structure, as well as the outer surfaces of the secondary crowns, were sandblasted with aluminum oxide (3 bar pressure, grain size 50 μm), creating a micro-rough surface. Thereafter, all specimens were placed in an ultrasonic bath for 5 min and, following air drying, weighed on a balance with a precision of 1 μg (R200D; Sartorius AG, Göttingen, Germany).

**Fig. 3:**
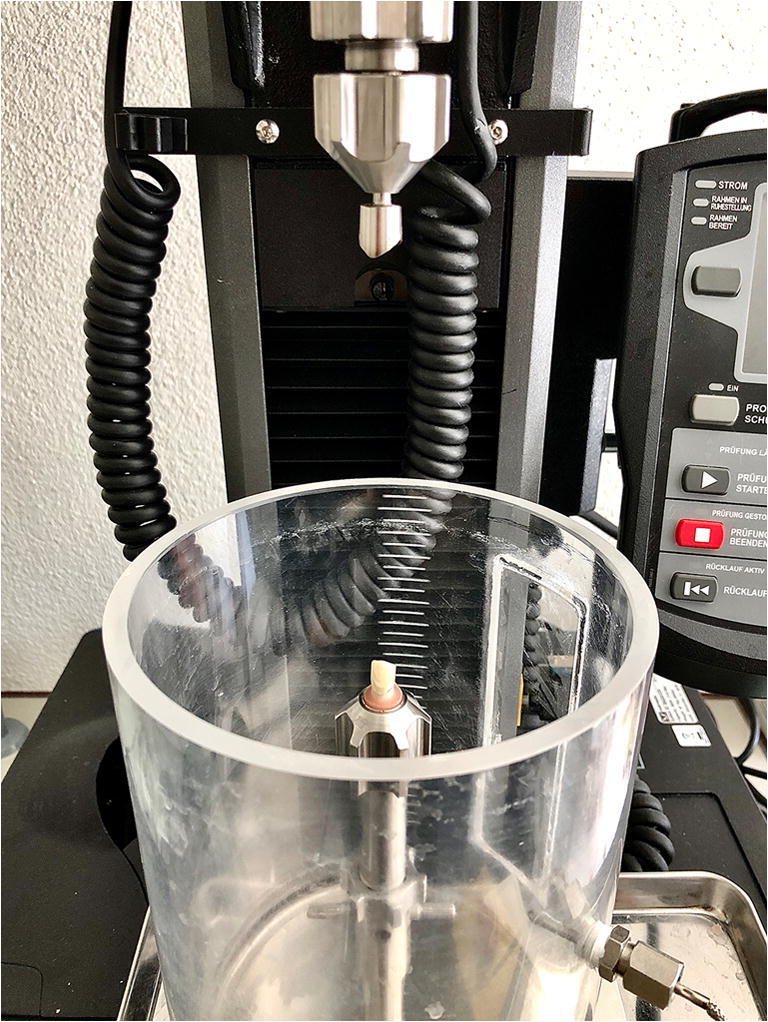
Specimen positioning with the cemented primary crown in the test device before luting of the secondary crown in the tertiary structure, which was positioned in the upper collet

The primary crowns were luted to the artificial polyurethane teeth using a chemically polymerizing PMMA (Paladur®). After polymerization, the base plates, including the specimens with the primary crowns, were mounted in the test device, and the secondary crowns were placed on the primary crowns in their final position. The base plate mounts of the test device were identical to those of the customized base plate manufacturing device, guaranteeing exact specimen repositioning (Fig. 3). The tertiary structure was filled with chemically polymerizing PMMA resin (Paladur®), fixated in the top collet of the measuring device, and moved downwards until the secondary crown was completely covered. After the polymerization process, the top collet, including the tertiary structure with the secondary crown, was moved upwards, and all excess resin was removed.

### Cyclic loading

Cyclic connections and disconnections were performed 10,000 times, simulating 10 years of use, assuming denture insertion and removal three times a day. The cycles were performed under wet conditions (distilled water) at a temperature of 37°C in a temperature-controlled bath. The crosshead speed was set to 1 mm/s while moving downward and 0.5 mm/s while moving upward. The secondary crowns were connected to the primary crowns with a force of 40 N, which was held for 1 s. The secondary crowns were then disconnected, and the maximum forces during disconnection were recorded in Newtons (N) by the test device. The vertical distance for each cycle was 3 mm. For the subsequent analyses, the maximum disconnection forces of cycles 10, 100, 500, 1000, 2000, 3000, 4000, 5000, 6000, 7000, 8000, 9000, and 10,000 were considered, but the data of every cycle were recorded. Data from the first 10 cycles were omitted to ensure stable testing conditions for all specimens. After finishing the cyclic connections, the primary and secondary crowns were removed from the specimens and the tertiary structure, respectively, using a Bunsen burner. All PMMA remnants on the crowns were carefully removed at 3.5-fold magnification, and the weight was assessed using the same high precision balance from the start of the experiment.

### Statistical analysis

A power analysis could not be performed due to the absence of reference data. Nevertheless, a higher sample size (*n* = 10 per group) relative to similar in vitro studies on double crowns was chosen [19–21]. For assessing the outcome of variable retention and wear (relative weight loss), a nonparametric, repeated measures ANOVA by Brunner and Langer [22], with the factors time (number of cycles, as repeated measurements) and material, was performed. Post hoc exact Mann–Whitney tests were performed to compare the retention forces and weight losses of all materials with each other after 10, 1000, 5000, and 10,000 loading cycles simulating baseline, 1, 5, and 10 years of use, respectively. Effect values for materials were estimated as the median difference (including 95% confidence intervals [CIs]) with respect to the baseline category PA/PA (control). To assess changes in retention forces over time within each material, exact Wilcoxon signed-rank tests were performed. Here, the effect values for cycles were estimated by the Hodges–Lehmann median (including 95% CIs) with respect to the baseline category of 10 loading cycles. To compare the behavior over time between each pair of materials, nonparametric repeated-measures ANOVAs were again utilized. Throughout the analysis, *p* values smaller than 0.05 were considered statistically significant. All analyses were performed with the statistics software R version 3.5.0 [23].

## Results

No fractures or decementations were observed during the procedure. Materials and loading cycles showed significant impacts on both retention force and relative weight loss (all *p* < 0.0001), including significant interaction (all *p* < 0.0001) according to the repeated measures nonparametric ANOVA.

### Evolution of retention forces within the groups

**Fig. 4:**
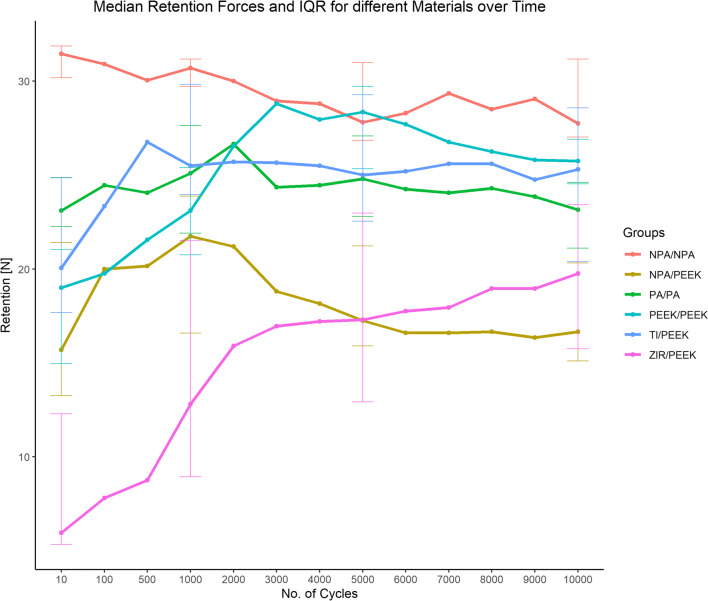
Change of retention forces for each material combination over time. “No. of Cycles” refers to the number of insertion–separation cycles; retention was consecutively tested for 10–10,000 cycles. PA = precious alloy, NPA = non-precious alloy, PEEK = polyether ether ketone, TI = titanium, and ZIR = zirconia

An overview of the retention forces during all cycles is depicted in Fig. 4. All materials were evaluated separately, comparing the retention forces at the beginning and the end of six cycling intervals: the initial year of use (I) = cycle 10 vs. 1000; the initial 5 years of use (II) = cycle 10 vs. 5000; the initial 10 years of use (III) = cycle 10 vs. 10,000; the second to the fifth year of use (IV) = cycle 1000 vs. 5000; the second to the tenth year of use (V) = cycle 1000 vs. 10,000; and the sixth to the tenth year of use (VI) = cycle 5000 vs. 10,000.

A statistically significant increase in retention forces was observed for test group 1 (ZIR/PEEK) for all intervals (I–V: *p* = 0.002; VI: *p* = 0.004). As for test group 2 (TI/PEEK), the intervals I (*p* = 0.01) and II (*p* = 0.02) showed increases, whereas a significant decrease of retention forces was found over interval V (*p* = 0.04). In test group 3 (PEEK/PEEK), retention forces rose significantly over all intervals except intervals V and VI (I–III: *p* = 0.002; IV: *p* = 0.004). Significant differences could be found for test group 4 (NPA/PEEK) in the intervals IV (*p* = 0.01), V (*p* = 0.004), and VI (*p* = 0.03), strictly decreasing after 1000 cycles. For test groups 5 (NPA/NPA) and 6 (PA/PA), only one interval presented a significant decrease in retention force over time. In group 5 (NPA/NPA), this was interval IV (*p* = 0.049); and in group 6 (PA/PA), it was interval VI (*p* = 0.008). Table 4 lists an overview of the estimated differences between baseline and 1000, 5000, and 10,000 cycles, including 95% confidence intervals (CIs) and separated for all materials.

**Table 4:** Overview of significantly different retention forces between applied material combinations at specific loading cycles. *PA*, precious alloy; *NPA*, non-precious alloy; *PEEK*, polyether ether ketone, *TI*, titanium; *ZIR*, zirconia

Number of loading cycles	Material combination 1	Material combination 2	*p* value
10	PA/PA	NPA/NPA	< 0.0001
	PA/PA	PEEK/PEEK	0.009
	PA/PA	ZIR/PEEK	< 0.0001
	NPA/NPA	NPA/PEEK	0.0002
	NPA/NPA	PEEK/PEEK	< 0.0001
	NPA/NPA	TI/PEEK	0.0007
	NPA/NPA	ZIR/PEEK	< 0.0001
	NPA/PEEK	ZIR/PEEK	0.006
	PEEK/PEEK	ZIR/PEEK	0.004
	TI/PEEK	ZIR/PEEK	0.0007
1000	PA/PA	NPA/NPA	0.003
	PA/PA	ZIR/PEEK	0.004
	NPA/NPA	NPA/PEEK	< 0.0001
	NPA/NPA	PEEK/PEEK	0.0007
	NPA/NPA	TI/PEEK	0.03
	NPA/NPA	ZIR/PEEK	< 0.0001
	NPA/PEEK	TI/PEEK	0.01
	PEEK/PEEK	ZIR/PEEK	0.009
	TI/PEEK	ZIR/PEEK	0.0005
5000	PA/PA	NPA/NPA	0.006
	PA/PA	ZIR/PEEK	0.045
	NPA/NPA	NPA/PEEK	< 0.0001
	NPA/NPA	ZIR/PEEK	0.0001
	NPA/PEEK	PEEK/PEEK	0.003
	NPA/PEEK	TI/PEEK	0.0007
	PEEK/PEEK	ZIR/PEEK	0.002
	TI/PEEK	ZIR/PEEK	0.01
10,000	PA/PA	NPA/NPA	0.002
	NPA/NPA	NPA/PEEK	< 0.0001
	NPA/NPA	PEEK/PEEK	0.045
	NPA/NPA	ZIR/PEEK	0.005
	NPA/PEEK	PEEK/PEEK	0.0001
	NPA/PEEK	TI/PEEK	0.003

### Evolution of retention forces across the groups

With respect to the parameter “material,” significant differences of all cumulative loading cycles were found when the control group (PA/PA) was compared to groups 1 (ZIR/PEEK) and 3 (PEEK/PEEK) (*p* < 0.001). Comparisons between the control group and groups 2 (TI/PEEK, *p* = 0.15), 4 (NPA/PEEK; *p* = 0.47), and 5 (NPA/NPA, *p* = 0.06) revealed no statistically significant differences, demonstrating similar evolution of retention forces. An overview of retention force differences at 10, 1000, 5000, and 10,000 cycles, comparing the test groups to the control group, is presented in Table 5.

**Table 5:** Overview of differences in the weight loss of primary and secondary crowns comparing all applied materials. *PA*, precious alloy; *NPA*, non-precious alloy; *PEEK*, polyether ether ketone; *TI*, titanium; *ZIR*, zirconia

Material combination 1	Material combination 2	*p* value “Primary”	*p* value “Secondary”
PA/PA	NPA/NPA	< 0.0001	< 0.0001
PA/PA	NPA/PEEK	1	0.12
PA/PA	PEEK/PEEK	< 0.0001	0.001
PA/PA	TI/PEEK	< 0.0001	< 0.0001
PA/PA	ZIR/PEEK	< 0.0001	0.001
NPA/NPA	NPA/PEEK	0.014	< 0.0001
NPA/NPA	PEEK/PEEK	0.053	0.005
NPA/NPA	TI/PEEK	0.66	0.02
NPA/NPA	ZIR/PEEK	0.06	0.91
NP/PEEK	PEEK/PEEK	0.008	0.0001
NP/PEEK	TI/PEEK	0.01	< 0.0001
NPA/PEEK	ZIR/PEEK	0.008	< 0.0001
NPA/PEEK	TI/PEEK	0.48	0.54
PEEK/PEEK	ZIR/PEEK	0.53	0.02
TI/PEEK	ZIR/PEEK	0.55	0.04

### Wear

**Fig. 5:**
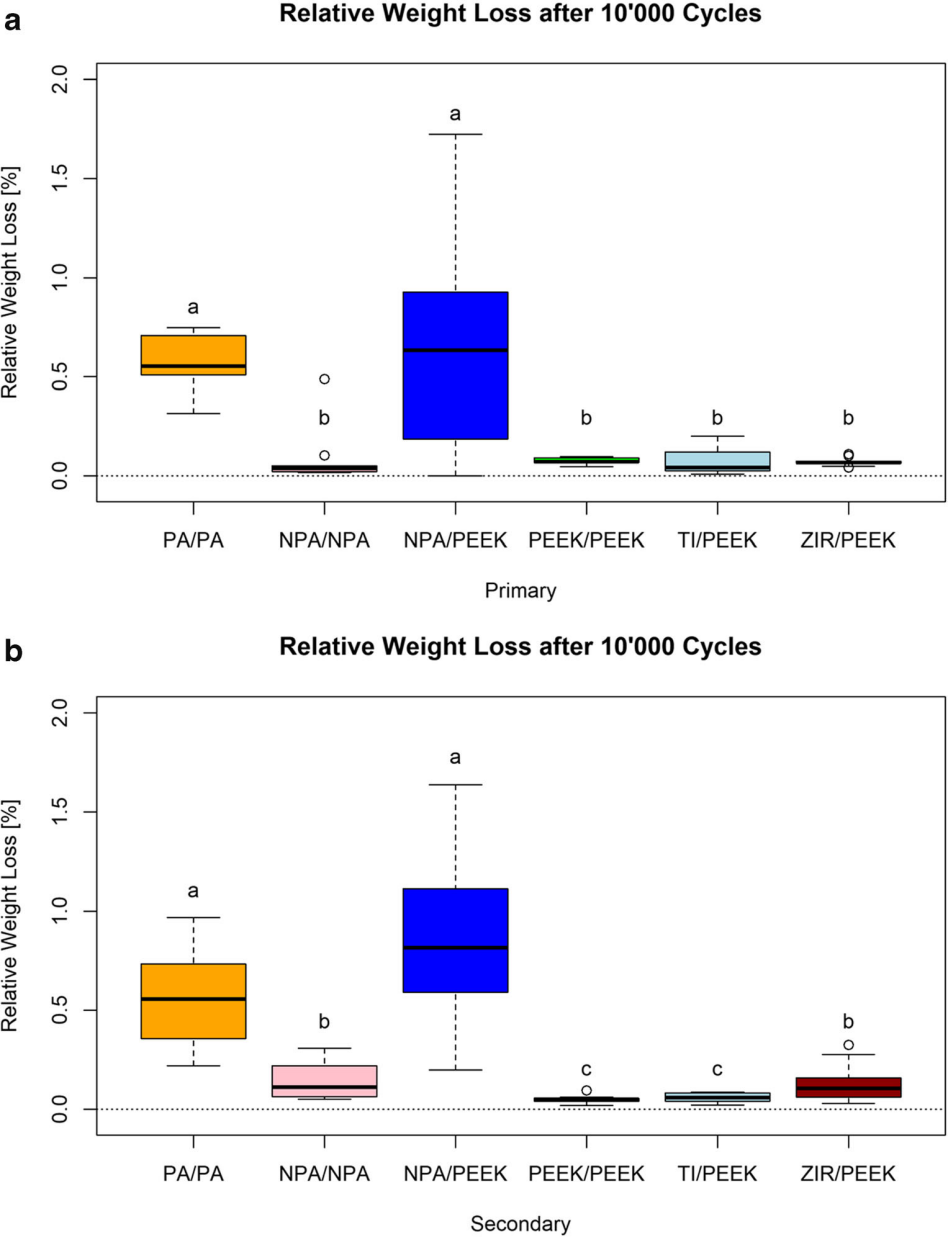
**a** Relative weight loss (%) in primary crowns in all applied material combinations; PA = precious alloy, NPA = non-precious alloy, PEEK = polyether ether ketone, TI = titanium, and ZIR = zirconia. Alphabetic superscripts indicate significant differences between the groups. **b** Relative weight loss (%) in secondary crowns in all applied material combinations; PA = precious alloy, NPA = non-precious alloy, PEEK = polyether ether ketone, TI = titanium, and ZIR = zirconia. Alphabetic superscripts indicate significant differences between the groups

Wear was evaluated by the relative weight loss of the primary and secondary crowns during 10,000 connection and disconnection cycles. The weighing of each primary and secondary crown was performed before and after the cyclic loading. Figure 5 provides an overview of the relative weight loss of the primary and secondary crowns. The highest relative weight loss was found in groups 4 (NPA/PEEK) and 6 (PA/PA). However, comparing the control to the test groups, this loss was not significant for group 4 (for which we observed the greatest change in weight across all groups), neither in the primary (*p* = 1) nor in the secondary crowns (*p* = 0.12).

## Discussion

The current study compared the evolution of retention forces between primary and secondary telescopic crowns milled from various materials to the retention forces of cast precious metal alloy primary and secondary crowns, simulating 10 years of denture insertion and removal. Based on our observations, the null hypothesis of equal retention forces was rejected. Statistically significant differences for the parameters’ material, cycling, interaction, and wear could be demonstrated.

CAD/CAM technology is characterized by its high precision and is currently used to mill and fabricate telescopic crowns [24]. Furthermore, an increase in efficiency is attributed to the use of CAD/CAM processes, which avoids casting technology-associated errors [25–27]. The strength of this study lies primarily in the uniform design for the primary and secondary crowns, including the control group in which the primary crown was initially fabricated from a millable wax. Only the secondary crowns in the control group were made using a conventional metal casting, which is still the gold standard for secondary crown fabrication.

Canines are among the most commonly used teeth for anchoring telescopic dentures. Therefore, in this study, we decided to use an artificial canine to design the telescopic crowns. However, telescopic prostheses are rarely fabricated on single teeth, which is probably the biggest limitation in this study. Nevertheless, previous studies have tested the retention forces of double crowns on single teeth to model the effects of parameters such as the crown taper, material, or the length of the retentive portion on retention force [19–21]. Another limitation of our approach is that horizontal and extra-axial forces, which are known to accelerate wear behavior, were not applied [28]. In addition, both the primary and secondary crowns were fabricated with lower material layer thicknesses than described in the literature [19–21]. The idea behind this was to evaluate whether these new material combinations would survive 10 years of simulated insertion and removal even in these reduced layer thicknesses and thus potentially eliminate one of the main limitations of double crowns, which is over contouring [29]. Since no major damage to the crowns was observed, it is unlikely that the thin layer thicknesses had any effect on the retention forces due to the complete coverage of the crowns in a tertiary framework and the associated prevention of bending up.

Retention forces have been tested under moist conditions in previous studies on telescopic crowns. The moist conditions are necessary to generate hydraulic adhesion between primary and secondary crowns [30]. Both distilled water [31] and artificial saliva [32] have been used in previous studies to create a moist environment. Our preliminary tests showed no difference between the two media; therefore, we decided to use distilled water for simplicity. Cyclic loading under wet conditions enables the formation of negative pressure in the occlusal gap while loosening the secondary crown. The compensation occurs via a delayed salivary flow (here, distilled water) in the area of the parallel surfaces between the primary and secondary crowns. The resulting flow resistance (Hagen–Poiseuille law) between the contact surfaces increases the adhesion, especially in very thin capillary-like gaps, such as can be found between the primary and secondary crowns due to the CAD/CAM milling process [33]. This may explain why the retention forces in this study are significantly higher than the 5–10 N described as ideal in the literature, especially considering that the retention forces in the control group were in a similar high range. Such comparatively high retention forces have also been demonstrated in previous in vitro studies on retention forces under moist conditions [19, 31]. Using the present telescopic-crown materials under in vivo conditions, the retention forces should be tested intraorally, and if exceeding the desired forces, manual polishing of the primary crowns’ outer and/ or secondary crowns’ inner surfaces may be applied. Furthermore, increasing the offset between primary and secondary crowns, increasing the taper, or reducing the length of the retentive portions may represent further options during the CAD/CAM process to decrease retention forces [19–21, 32, 34].

All applied materials demonstrated a significant change in retention force over at least one of the evaluated time intervals. However, after ten simulated years of denture insertion and removal, no significant changes in retention forces relative to baseline were observed in groups 2 (TI/PEEK), 4 (NPA/PEEK), 5 (NPA/NPA), and 6 (PA/PA). The retention forces in group 1 (ZI/PEEK) increased constantly and significantly over the entire study period, whereas the retention forces in group 3 (PEEK/PEEK) increased only during the first five simulated years, remaining constant thereafter. The only two groups that demonstrated significantly higher retention forces after 10 years were those which did not include a metal alloy primary crown. In general, an increase in retention forces is often seen in the early stages of testing various retentive elements for prostheses. This is attributed to initial plastic deformation and an increase in surface roughness [21, 35]. The outer surfaces of the primary crowns and the inner surfaces of the secondary crowns are prone to elastically reversible deformations and plastically irreversible deformations during insertion, removal, and chewing movements. Consequently, a possible explanation for these developments of the retention forces could be material-specific deformation or wear. In general, it can be assumed that an increase in roughness leads to an increase in retention force. In addition, the mechanical adaptation of the primary and secondary crowns at their interfaces may play a role in the increase [31]. However, this increase in retention force only continues until a critical limit of plastic deformation of at least one of the two crowns is reached. If this limit is exceeded, this leads to a decrease in the retention force since the interface between the primary and secondary crowns is no longer congruent and the desired static friction can no longer occur. The fact that the two groups in which retention forces increased were among the groups with the lowest relative weight loss supports the theory that material wear in these groups did not reach the critical limit to precipitate a decrease in retention force.

At the beginning (10 cycles) and at the end of the cyclic connections and disconnections (10,000 cycles), significantly different retention forces between most of the material groups were observed, which was also demonstrated in a previous study applying similar material combinations [31]. Comparing the evolution of retention forces, no significant differences between the control group and group 2 (TI/PEEK, 4 (NPA/PEEK) or 6 (NPA/NPA) were found, which means that all groups including at least one metal part demonstrated similar retention behavior. Since the material combination PA/PA has been shown to be reliable, it seems advantageous to retain at least one metallic component in any possible material combination for telescopic crowns. Creating an optimum surface morphology using CAD (in addition to the precision of the milling process), the selected fit parameters, ideal tools, and the milling path strategy are decisive [15]. An improved fit with a consequently narrower joint gap between the primary and secondary crowns may be achieved using digital fabrication technologies. However, the knowledge regarding how an increased fit between primary and secondary crowns using different materials affects the development of retention forces, as well as the wear resistance, is very limited. Our results show that both the retention behavior and the wear were very different despite the standardized design of the primary and secondary crowns. The consequences of this could be that for each material combination, the optimal design must be separately determined and that earlier results on precious metal telescopic crowns cannot simply be transferred to these new material combinations.

Wear of the secondary and especially primary telescopic crowns presents a major challenge for clinicians. While secondary crowns may be replaced with relative ease, replacing primary crowns on teeth requires the destruction of the crown, which is accompanied by potential damage to the remaining tooth structure. Therefore, one of the main requirements for double-crown systems is to choose a wear-resistant material, especially for the primary, but also for the secondary telescopic crown. In terms of wear resistance, the material combinations NPA/NPA, ZIR/PEEK, PEEK/PEEK, and TI/PEEK demonstrated more favorable outcomes relative to the control group. While ZIR/PEEK and PEEK/PEEK showed a constant increase in retention forces, the evolution of retention forces in the material combinations TI/PEEK and NPA/NPA were similar to the control group and may therefore be recommended for clinical application. The suitability of these material combinations has already been demonstrated in previous in vitro studies, with two (NPA/NPA) or four telescopic crowns (TI/PEEK) per denture, respectively [9, 31]. Although a significant decrease of retention forces (36.17%) has been reported for casted NPA/NPA telescopic crowns after simulated 10 years of use, a recent in vitro study demonstrated a significantly lower loss of retention in milled compared to casted NPA/NPA double crowns, confirming the results of the present study [18]. The difference between the casted and milled groups was mainly attributed to the higher accuracy and the lower surface roughness of the milled group [18]. The material combination NPA/NPA has also been evaluated in a retrospective clinical study and while the authors of that study did not observe a difference in denture survival, the abutment survival rate was significantly higher in the control group using PA/PA telescopic crowns [36]. However, the manufacturing technique of the NPA/NPA crowns has not been reported in that specific study.

Future in vitro studies should evaluate the effects of various designs, such as the taper or the gap size between primary and secondary crowns milled from promising, wear-resistant material combinations, e.g., TI/PEEK. The evolution of retention forces in such studies should be evaluated in a clinically more relevant setup with four telescopic crowns in a strategically beneficial distribution across the jaw. Furthermore, in addition to retention forces, other potential problems, such as the cementation of these new materials or the development of surface roughness due to wear and associated plaque adhesiveness, need to be investigated [18, 37]. The results of those in vitro studies should be completed by in vivo studies focusing specifically on abutment survival.

## Conclusion

The materials used for milling primary and secondary telescopic crowns have an effect on the absolute retention forces, the evolution of retention forces over time, and the resistance to wear, even if the same crown design is chosen. The material combinations titanium/PEEK and non-precious alloy/non-precious alloy can be recommended for future research. The evolution of retention forces with these combinations was comparable to precious alloy/precious alloy telescopic crowns, but the wear was even smaller. The existing knowledge regarding telescopic crowns consisting of precious metal alloy primary and secondary crowns cannot be directly transferred to other material combinations, even if the same design is used for crown fabrication.

## References

[1] Bohnenkamp DM (2014). Removable partial dentures: clinical concepts. Dent Clin N Am.

[2] Campell SD, Cooper L, Craddock H, Hyde TP, Nattress B, Pavitt SH, Seymour DW Removable partial dentures: the clinical need for innovation. J Prosthet Dent 118:273–280 10.1016/j.prosdent.2017.01.008 28343666

[3] Melilli D, Davì G, Messina P, Scardina GA (2017). Tooth-implant connection in removable denture. A review. Minerva Stomatol.

[4] Wagner B, Kern M (2000). Clinical evaluation of removable partial dentures 10 years after insertion: success rates, hygienic problems, and technical failures. Clin Oral Investig.

[5] Bayer S, Steinheuser D, Grüner M, Keilig L, Enkling N, Stark H, Mues S (2009). Comparative study of four retentive anchor systems for implant supported overdentures – retention force changes. Gerodontology.

[6] Hakkoum MA, Wazir G (2018). Telescopic denture. Open Dent J.

[7] Stancis I, Jelenkovic A (2008). Retention of telescopic denture in elderly patients with maximum partially edentulous arch. Gerodontology.

[8] Weaver JD (1989). Telescopic copings in restorative dentistry. J Prosthet Dent.

[9] Arnold C, Hey J, Setz JM, Boeckler AF, Schweyen R (2018). Retention force of removable partial dentures with different double crowns. Clin Oral Investig.

[10] Yi YJ, Cho LR, Park CJ (2003). Cause of technical failures of conical crown-retained denture (CCRD): a clinical report. J Korean Acad Prosthodont.

[11] Glantz PO (1984). Intraoral behaviour and biocompatibility of gold versus non precious alloys. J Biol Buccale.

[12] Skirbutis G, Dzingutė A, Masiliūnaitė V, Šulcaitė G, Žilinskas J (2018). PEEK polymer’s properties and its use in prosthodontics. A review. Stomatologija.

[13] Merk S, Wagner C, Stock V, Eichberger M, Schmidlin PR, Roos M, Stawarczyk B (2016). Suitability of secondary PEEK telescopic crowns on zirconia primary crowns: the influence of fabrication method and taper. Materials.

[14] Stock V, Schmidlin PR, Merk S, Wagner C, Roos M, Eichberger M, Stawarczyk B (2016). PEEK primary crowns with cobalt-chromium, zirconia and galvanic secondary crowns with different tapers-a comparison of retention forces. Materials.

[15] Wagner C, Stock V, Merk S, Schmidlin PR, Roos M, Eichberger M, Stawarczyk B (2018). Retention load of telescopic crowns with different taper angles between cobalt-chromium and polyetheretherketone made with three different manufacturing processes examined by pull-off test. J Prosthodont.

[16] Heimer S, Schmidlin PR, Roos M, Stawarczyk B (2017). Surface properties of polyetheretherketone after different laboratory and chairside polishing protocols. J Prosthet Dent.

[17] Bathala L, Majeti V, Rachuri N, Singh N, Gedela S (2019). The role of polyether ether ketone (PEEK) in dentistry – a review. J Med Life.

[18] Luft V, Pospiech P, Schurig A, Schmitter M (2021). In vitro investigations on retention force behavior of conventional and modern double crown systems. Dental Mater.

[19] Schwindling FS, Stober T, Rustemeier R, Schmitter M, Rues S (2016). Retention behavior of double-crown attachments with zirconia primary and secondary crowns. Dent Mater.

[20] Engels J, Schubert O, Güth JF, Hoffmann M, Jauering C, Erdelt K, Stimmelmayr M, Beuer F (2013). Wear behavior of different double-crown systems. Clin Oral Investig.

[21] Besimo CH, Graber G, Flühler M (1996). Retention force changes in implant-supported titanium telescope crowns over long-term use in vitro. J Oral Rehabil.

[22] Brunner E, Domhof S, Langer F (2012) nparLD: nonparametric analysis of longitudinal data in factorial experiments, R package version 2.1

[23] R Core Team (2019). R: a language and environment for statistical computing. R Foundation for Statistical Computing, Vienna, Austria. URL https://www.R-project.org/.

[24] Arnold C, Schweyen R, Boeckler A, Hey J (2020). Retention force of removable partial dentures with CAD-CAM-fabricated telescopic crowns. Materials.

[25] Shimakura M, Nagata T, Takeuchi M, Nemoto T (2008). Retentive force of pure titanium konus telescope crowns fabricated using CAD/CAD system. Dent Mater J.

[26] Arnold C, Hey J, Schweyen R, Setz JM (2018). Accuracy of CAD-CAM-fabricated removable partial dentures. J Prosthet Dent.

[27] Yoshikawa Y, Torii K, Tanaka M (2019). Influence of the number of insertions and removals of telescopic zirconia/alumina crowns on retentive force and settling. Dent Mater.

[28] Bayer S, Zuziak W, Kraus D, Keilig L, Stark H, Enkling N (2010). Conical crowns with electroplated gold copings: retention force changes caused by wear and combined off-axial load. Clin Oral Implants Res.

[29] Beschnidt SM, Chitmongkolsuk S, Prull R (2001). Telescopic crown-retained removable partial dentures: review and case report. Compend Contin Educ Dent.

[30] Beuer F, Edelhoff D, Gernet W, Naumann M (2010). Parameters affecting retentive force of electroformed double-crown systems. Clin Oral Investig.

[31] Elkabbany A, Kern M, Elkhadem AH, Wille S, Amer AA, Chaar MS (2020). Retention of metallic and non-metallic double-crown-retained mandibular overdentures on implants: an in-vitro study. J Prosthodont Res.

[32] Bayer S, Kraus D, Keilig L, Gölz L, Stark H, Enkling N (2012). Changes in retention force with electroplated copings on conical crowns: a comparison of gold and zirconia primary crowns. Int J Oral Maxillofac Implants.

[33] Nakagawa S, Torii K, Tanaka M (2017). Effects of taper and space setting of telescopic Ce-TZP/A crowns on retentive force and setting. Dent Mater.

[34] Fischer CAI, Ghergic DL, Vranceanu DM, Ilas SA, Comaneanu RM, Baciu F, Cotrut CM (2020). Assessment of force retention between milled metallic and ceramic telescopic crowns with different taper angles used for oral rehabilitation. Materials (Basel).

[35] Yabul A, Dayan C, Geckili O, Bilhan H, Tuncer N (2018). Evaluation of volumetric wear of abutments on the retention loss of ball attachment systems in implant-retained overdentures: an in vitro study. Clin Implant Dent Relat Res.

[36] Zierden K, Kurzrock L, Wöstmann B, Rehmann P (2018). Nonprecious alloy vs precious alloy telescopic crown-retained removable partial dentures: survival and maintenance needs. Int J Prosthodont.

[37] Stawarczyk B, Taufall S, Roos M, Schmidlin PR, Lümkemann N (2018). Bonding of composite resins to PEEK: the influence of adhesive systems and air-abrasion parameters. Clin Oral Investig.

